# Management of PEComas: A Review of the Role of Radiotherapy

**DOI:** 10.3390/cancers18091388

**Published:** 2026-04-27

**Authors:** Kristina Nesterova, Reinhardt Krcek, Abha A. Gupta, Peter W. M. Chung

**Affiliations:** 1 Arthur J.E. Child Comprehensive Cancer Centre, Department of Medical Oncology, Calgary, AB T2N 5G2, Canada; 2 University of Calgary, Calgary, AB T2N 1N4, Canada; 3 Department of Radiation Oncology, University Hospital Zurich, University of Zurich, 8091 Zurich, Switzerland; reinhardt.krcek@usz.ch; 4 University of Toronto, Toronto, ON M5S 3H2, Canada; abha.gupta@uhn.ca (A.A.G.); peter.chung@uhn.ca (P.W.M.C.); 5 Division of Hematology/Oncology, Hospital for Sick Children, Toronto, ON M5G 1X8, Canada; 6 Division of Medical Oncology, Princess Margaret Cancer Centre, University Health Network, Toronto, ON M5G 2M9, Canada; 7 Radiation Medicine Program, Princess Margaret Cancer Centre, University Health Network, Toronto, ON M5G 2M9, Canada

**Keywords:** PEComa, radiotherapy, local control, rare sarcoma, ultra-rare sarcoma

## Abstract

**Background/Objectives**: Malignant PEComa is a rare sarcoma subtype and usually represents PEComa-NOS (not otherwise specified), one of the several entities of the PEComa family. Surgery is the primary treatment for localized disease; chemotherapy is used mainly for metastatic or unresectable cases. Radiotherapy (RT) may be considered in selected cases; however, its role remains unclear due to the rarity of the disease and limited radiotherapy-specific studies. **Methods**: This is a descriptive literature review of a limited number of reports on RT use in PEComa. Descriptive statistics were used to summarize reported case characteristics and outcomes. **Results**: We identified 28 publications reporting 33 cases. In neoadjuvant settings, there was a significant local response to RT in one case. In other neoadjuvant cases, although quantitative response assessments were not reported, most showed no recurrence during follow-up, with the longest follow-up at 34 months, suggesting that a possible benefit in local disease control may exist. In the adjuvant setting, some reports described prolonged disease-free survival following RT, though the lack of direct comparisons between surgery with versus without RT and heterogeneous follow-up periods limit definitive conclusions. In selected metastatic cases, palliative RT achieved notable local responses, potentially contributing to durable local control. **Conclusions**: In conclusion, although the only available data on RT in PEComas come from case studies with overall heterogeneous management approaches, RT has shown some potential as a therapeutic option across neoadjuvant, adjuvant, and palliative settings, warranting further dedicated clinical studies.

## Simple Summary

Malignant PEComa is an ultra-rare and aggressive subtype of sarcoma. Current treatment options mainly include surgery for localized, and systemic therapy for metastatic disease, while the role of radiotherapy (RT) remains unclear in all settings. This review summarizes published clinical reports on the use of RT for PEComa and describes treatment outcomes. Although the available evidence is limited to heterogeneous case reports and often includes additional therapies that make interpretation difficult, a considerable number of cases demonstrated local disease control and/or sustained disease stability after RT, suggesting that RT may be a possible therapeutic contributor and supporting the need for dedicated clinical studies evaluating RT in PEComa.

## 1. Introduction

Perivascular epithelioid cell neoplasm (PEComa) is an ultra-rare mesenchymal tumor that arises from the perivascular epithelioid cells (PEC) and may occur in any part of the body [1]. The entity was first formally described in 1992 when perivascular epithelioid cells were found in a clear cell tumor of the lung (CCTL) as well as kidney angiomyolipoma (AML) and were characterized by strict association with the vessel smooth muscle walls and exhibited unusual histological features resembling both melanocytes and muscle cells [2]. Later, Zamboni et al. described a tumor found in the pancreas with similar pathohistological and immunochemical characteristics and the term PEComa was subsequently proposed to encompass this ‘family’ of tumors [3]. The World Health Organization included PEComa in its 2002 classification of diseases [4].

Historically, the PEComa family of tumors comprises angiomyolipomas (AML), lymphangioleiomyomatosis (LAM), clear cell sugar tumor (CCST), and PEComa not otherwise specified (PEComa-NOS). The first three subtypes are usually considered as noncancerous and are not included in this review. The term ‘PEComa’ is typically attributed to PEComa-NOS, the histopathology of which can be classified as benign, uncertain malignant potential (UMP), and malignant. Malignant classification is based on six high-risk criteria, tumor size ≥ 5 cm, infiltrative growth pattern, high-nuclear-grade cellularity, mitotic rate > 1/50 high-power fields (HPF), necrosis, and vascular invasion, with two or more criteria required to be defined as malignant. PEComas with fewer malignant histological features, so-called uncertain malignant potential (UMP), may also show aggressive behavior, extensive metastases and a poor prognosis [5]. Importantly, given the rarity of PEComa-NOS, the risk of misclassification and grade misassignment remains high, as for many mesenchymal tumors/sarcomas; for this reason, centralized histopathological review in specialized sarcoma referral centers is strongly recommended [6].

Primary surgical excision with negative margins is crucial in treating PEComas [7,8]. With respect to pharmacotherapy, there is currently no clear consensus on the use of neoadjuvant or adjuvant drug-based regimens. Pharmacological treatment is often reserved for progressive or inoperable disease, most frequently using mTOR inhibitors [9,10,11]. Conventional systemic chemotherapy can be used although its objective response rate (ORR) is typically lower compared to that of mTOR inhibitors [12,13]. Other medical therapies, including Vascular Endothelial Growth Factor Receptor (VEGFR) and Programmed Cell Death Protein 1 (PD-1) inhibitors may be considered in cases of mTOR inhibitor resistance [14,15,16].

Another important clinical question that remains unanswered is the role of radiotherapy (RT) in the management of PEComa in the perioperative (neoadjuvant, adjuvant), palliative, and curative settings. Malignant PEComas exhibit features such as a high mitotic index and rich vascularization, which may be associated with increased sensitivity to radiation [17]. This might support the use of radiotherapy as a therapeutic option for patients with PEComas. This study aimed to review available data on radiotherapy in the management of PEComa to enhance the understanding of its application in these tumors.

## 2. Materials and Methods

The literature search started in April 2024 with a final update of the literature in May 2025 using different queries on the PubMed database. The following search terms, restricted to the title and/or abstract, were used: “PEComa” OR “Perivascular Epithelioid Cell Tumor” AND “treatment”; “PEComa” OR “Perivascular Epithelioid Cell Tumor” AND “radiotherapy”; “PEComa” OR “Perivascular Epithelioid Cell Tumor” AND “radiation therapy”; PEComa” OR “Perivascular Epithelioid Cell Tumor” AND “radiation”.

The selection criteria included case reports, case series, and clinical trials involving patients with malignant and uncertain malignant potential PEComa-NOS receiving RT in any setting (neoadjuvant, adjuvant, palliative, curative-intent) with clinical and/or radiological follow-up reported for at least 3 months. The literature search included studies published from 1990 until May 2025. Given the rarity of the tumor and the limited number of studies focused specifically on RT for these tumors, detailed information on RT dosage was preferred but not mandatory for inclusion.

We excluded non-English articles and those focusing on PEComa subtypes other than NOS including AML, LAM, and CCST. We included PEComa cases classified as malignant or UMP, given the recognized overlap in pathological features between these categories since UMP tumors may demonstrate characteristics associated with malignancy such as mitotic activity, nuclear atypia or pleomorphism, or larger tumor size and may have increased risk of recurrence and progression [18,19].

Data collected from the included studies were age, gender, size of the primary tumor, Folpe criteria (tumor size, mitotic activity, nuclear grade/cellular atypia, necrosis and infiltrative growth pattern, if provided), Folpe risk category (if provided), site of the primary tumor, site of metastatic lesions, treatment(s) received, follow-up periods, and reported outcomes.

## 3. Statistical Analysis

As this is a descriptive literature review of a limited number of available reports, no inferential statistical analysis was performed. Descriptive statistics, including means, medians, and proportions, were used to summarize reported case characteristics and outcomes.

Given the number of studies and heterogeneous outcome descriptors, most of which did not apply strict RECIST criteria, individual case outcomes were reported, and disease courses were combined into a composite outcome for descriptive analysis grouped by RT setting. In neoadjuvant and adjuvant settings, this corresponded to no evidence of disease at last follow-up. In palliative settings, it included reported disease stability, local and/or systemic response, and/or symptom improvement after radiotherapy.

## 4. Results

**Figure 1:**
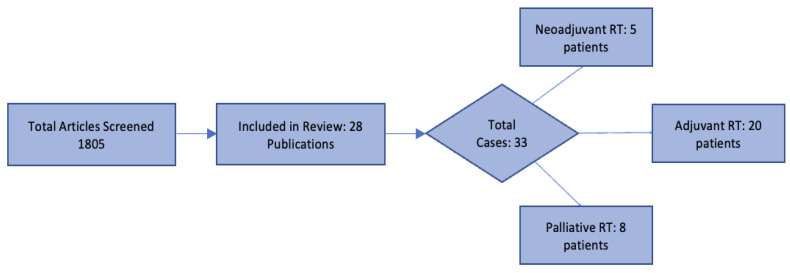
Study selection and distribution of RT settings in PEComa cases. A total of 1805 records were screened, with 28 publications (33 cases) included. Radiotherapy was delivered as neoadjuvant (*n* = 5), adjuvant (*n* = 20), and palliative (*n* = 8), with no cases treated with definitive RT intent.

A total of 1805 abstracts and any associated full-text articles were screened, of which 28 publications reporting 33 cases were included in this review. Of these, 5 patients received neoadjuvant RT, 20 received adjuvant RT, and 8 received palliative RT (Figure 1). There were no cases of definitive or curative-intent radiotherapy identified.

### 4.1. Neoadjuvant Radiotherapy

The number of reports on neoadjuvant radiotherapy for malignant PEComa treatment is limited. In total, we identified five cases that provided follow-up times and outcomes related to disease progression (Table 1) [20,21,22,23,24]. The median age was 49 years old, ranging from 28 to 68, with four of the five patients being female; the primary sites were musculoskeletal, oropharynx/neck, and liver. Follow-up times varied from 6 to 34 months with a median of 13 months. There was no evidence of disease recurrence (NED) reported in four out of five cases (80%).

Two cases included neoadjuvant chemotherapy in addition to RT [21,24]. One of these cases demonstrated progression of a neck PEComa despite treatment with mTOR inhibitors and conventional chemotherapy employing adriamycin and ifosfamide. This prompted the use of neoadjuvant RT, leading to a partial response and facilitating subsequent definitive surgical resection; volumetric change associated with RT was not provided. The patient remained NED at 6 months of follow-up [24]. In contrast, Osei et al. described a poor local response to neoadjuvant RT following neoadjuvant chemotherapy with doxorubicin and ifosfamide, initially resulting in tumor reduction but ultimately leading to a mild increase in tumor size before definitive surgical resection. The development of lung metastases had occurred by 13 months of follow-up [21].

Among all five cases, only one reported the exact dose of RT and associated volume reduction, where stereotactic RT of total 60 Gy in 8 fractions was applied to a liver PEComa lesion measuring 1280 cm^3^ and resulting in a 50% reduction by week 17 and NED at 21 months post-resection [23].

Notably, all reported lesions were larger than 5 cm, marked by high mitotic counts and pleomorphism with locations seemingly challenging for upfront surgery without attempting pre-operative tumor downsizing, which likely would have been facilitated by neoadjuvant RT. Also, it should be noted that two cases out of five did not demonstrate significant necrosis but had longer NED at 34 and 21 months of follow-up [22,23].

Overall, neoadjuvant RT appeared to lead to some degree of response, although exact volumetric measurements were lacking except for the case described above. In addition, two patients had follow-up periods of over 20 months and remained NED. This suggests that RT may have a potential role in durable disease control; however, the small number of cases prevents any firm conclusions. A detailed summary of the reported neoadjuvant cases is provided in Table 1, and a summary of RT treatments and clinical characteristics by treatment intent is presented in Appendix A.

### 4.2. Adjuvant Radiotherapy

There were 20 cases (16 publications) with adjuvant RT identified. The median age was 51 years old, ranging from 9 to 92, and females accounted for 80% of the cases. The primary sites were musculoskeletal, gynecological, skin, neck, retroperitoneal, adrenal gland, falciform ligament and mesenteric, with the most prevalent being gynecological sites: 10/20 cases (50%). Of the total number of cases, the median follow-up was 12 months (3–50 months). NED was reported in 15 of 20 cases (75%), and of these, combinations of high-risk histopathologic features (high mitotic activity and/or infiltrative growth and/or high nuclear grade and/or necrosis and/or vascular invasion) were described in 10 out of 15 cases (66%) [5,25,26,27,28,29,30,31,32,33]. One patient with a seemingly aggressive, locally recurrent, symptomatic pelvic PEComa following resection of an initial large malignant tumor (10 cm) remained NED at 50 months after re-resection, adjuvant RT and chemotherapy; however, no additional histopathologic features were described [32]. Of these ten cases, three involved cutaneous PEComas, while the remaining were musculoskeletal, oropharynx/neck, adrenal gland and gynecological [25,26,28]. The follow-up periods for these ten cases varied between 6 and 24 months with a median of 11 months and a mean follow-up period of around 13 months. Among these ten cases with NED, both RT and chemotherapy were administered in three cases involving a high-risk cutaneous lesion larger than 5 cm and uterine/pelvic PEComas with local lymph node involvement and large size [26,29,32]. One of these three cases, reported by Jeon et al., demonstrated NED at 18 months of follow-up [29].

Disease progression was observed in 5 out of 20 cases (25%) with four patients experiencing distant recurrence with metastasis to the lungs, liver, bones, small and large intestine, and abdominal wall and one patient experiencing local recurrence [5,34,35,36,37]. The median time to progression was 11 months (3–39 months). Primary tumor sites were falciform ligament (1/5), uterus (2/5), retroperitoneum (1/5), and mesentery (1/5). All five cases involved primary tumors of significant size ranging from 9 cm to 30 cm along with high-risk histopathologic features. Vascular invasion was reported in two cases, while necrosis and high mitotic activity were present in the majority of cases. Two patients received both adjuvant RT and chemotherapy for uterine PEComa, and one patient had concurrent adjuvant chemoradiation for mesenteric PEComa; progression time ranged between approximately 6 and 16 months [5,35,37].

Regarding RT doses, only 5 out of 20 cases reported total dose, which varied between 45 and 60 Gy [25,27,29,30,37]. These cases involved PEComas of the uterus, broad ligament, mesenteric region, adrenal gland, and skin. Among five cases, the mesentery PEComa, which received total 60 Gy of adjuvant concurrent RT with ifosfamide and adriamycin, recurred within 6 months [37].

Indications for adjuvant RT were not explicitly described in the presented case reports, except in three studies where it was administered for positive post-resection margins [30,36,38]. It might be assumed that tumor size in combination with other high-risk features and/or regional spread to lymph nodes would guide its use. In addition, there were a few cases in which adjuvant RT was used in patients with PEComa UMP, where concern regarding the unpredictable behavior of this tumor despite fewer high-risk characteristics might have driven the decision for adjuvant RT. All demonstrated NED at follow-up ranging between 6 and 30 months [5,38,39].

Overall, some of the presented cases of malignant PEComa with high-risk features demonstrated prolonged periods of disease control [27,28,29,30], with the longest NED observed at 50 months after complete resection of recurrent disease followed by adjuvant RT and chemotherapy [32]. However, given the heterogeneous follow-up periods, small sample size, lack of direct comparisons between adjuvant RT and no RT, and confounding from chemotherapy in some cases, conclusions regarding disease control remain difficult to draw. As for the reported progressed cases, a combination of large tumor size, intra-abdominal/pelvic primary location, high mitotic activity, and necrosis appeared to be associated with more unfavorable outcomes. In addition, among the cases that progressed, patients who received adjuvant RT and chemotherapy or concurrent chemoradiotherapy seemed to have a higher rate of progression (3 out of 8) compared to those receiving only adjuvant RT (2 out of 12). However, meaningful comparisons are limited as multimodality approaches are typically used in patients whose tumors exhibit multiple unfavorable features. These tumors were perhaps already predisposed to a more aggressive biological behavior, which may have warranted more intensive multimodality treatment. A detailed summary of adjuvant RT cases is provided in Table 2, and a summary of RT treatments and clinical characteristics by treatment intent is presented in Appendix A.

### 4.3. Metastatic Disease, Advanced/Unresectable PEComa and Palliative Radiotherapy

In total, eight case reports were identified that used palliative RT for PEComa metastases or inoperable disease [40,41,42,43,44,45,46]. The median age of patients was 46.5 years (range 19–75); and 5/8 (63%) were female. The primary PEComa sites were the uterus, pelvis, retroperitoneum, lung, and limbs, with one case of unknown primary tumor site. Follow-up after RT ranged between 4 and 48 months with a median follow-up time of 18 months. All reported primary PEComa lesions were above 5 cm with high-risk histologically malignant features including abundant necrosis.

Out of eight cases, one case experienced both local and systemic progression 10 months after pelvic consolidative radiotherapy and chemotherapy for residual, non-operable disease following resection [41]. De León et al. described two cases treated with palliative RT (13 Gy) for spinal metastases; however, no information on local control was provided. Both patients subsequently developed further disease progression involving additional spinal segments at 11 and 48 months [42].

Four cases described either disease stability or partial responses to palliative RT [40,43,44,45,46]. Separately, Lao et al. reported a case of metastatic malignant PEComa affecting the left femur for which the patient received palliative RT and was started on systemic chemotherapy; the patient was alive at 42 months, with no reported evidence of progression, although detailed response assessment and disease status were not provided [43].

Local RT responses were specifically highlighted in two cases. One reported a complete response by 4 months post-RT with 36 Gy (12 fractions) to a lung PEComa mass [40]. The other described a significant partial response to stereotactic body radiation therapy (SBRT) of 30 Gy (6 fractions) for a recurrent pelvic mass, along with an additional 24 Gy of SBRT delivered to a spine metastasis; the follow-up duration from treatment initiation was approximately 4 months [44]. However, the treatment also included a PD-1 inhibitor, tislelizumab, administered in the peri-radiotherapy period as well as granulocyte-macrophage colony-stimulating factor (GM-CSF), which was delivered concurrently with RT. Similarly, another patient with spinal metastasis treated with palliative radiotherapy alongside initiation of PD-L1 inhibitor therapy had stable disease for 12 months of follow-up [45]. Although positive RT response and/or RT-related local disease control is confounded by the use of immunotherapy in these cases, it does raise the question of a potential synergistic effect between radiotherapy and immunotherapy, which has been described in studies involving non-sarcoma malignancy yet may be potentially applicable to sarcoma [47]. Importantly, there are currently no standardized morphopathological, molecular, or genetic markers of radiosensitivity, nor validated RT-specific predictive models that can be used in clinical settings to explain variability in RT response in PEComa cases. This is particularly relevant given the historical perception that PEComa derives very limited benefit from radiotherapy [48,49]. In addition, higher RT doses were reported in cases demonstrating positive local responses [40,44]. However, the majority of case reports did not specify the exact palliative RT dose used, making interpretation of a dose-response relationship difficult.

Overall, both palliative radiotherapy and systemic therapy were reported in six out of eight cases, reflecting the standard approach for managing aggressive and metastatic PEComa. Although the individual contribution of RT versus systemic therapy to disease control is challenging to define, some cases demonstrated either prolonged disease stability during the follow-up period or local tumor regression after palliative RT. Similar to other scenarios included in this review, the small number of reports, heterogeneous follow-up periods, and lack of RT-focused details limit the ability to draw clear conclusions. Reported cases are summarized in Table 3 and a summary of RT treatments and clinical characteristics by treatment intent is presented in Appendix A.

## 5. Conclusions and Future Directions

Although the role of RT in the management of PEComa remains unclear, due to limited data in this ultra-rare disease, this review highlights the emerging evidence that RT might contribute to disease control in both the primary and metastatic setting. More dedicated, RT-focused clinical studies are needed to better define the therapeutic effect of RT in PEComa. As prospective research designs would most likely be limited by challenges in achieving adequate sample size, in this context, the conduct of RT-specific retrospective studies may provide significant insight into this area. RT-dedicated retrospective research could also explore potential synergistic effects between RT and systemic therapies used in PEComa, as well as possible associations between radiotherapy response and molecular profiles (e.g., mTOR pathway alterations versus TFE3 rearrangements).

Given the current lack of robust evidence, the management of PEComa, including the use of radiotherapy in any setting, would benefit from multidisciplinary discussions within and even across specialized sarcoma referral centers, including radiation oncologists, medical oncologists, surgical oncologists, pathologists, and other sarcoma specialists, to support individualized treatment strategies. In the absence of such an approach, management often relies on historically adopted, generalized treatment paradigms, which may not adequately address the complexity of PEComa and may lead to potentially effective treatment modalities such as radiotherapy being underutilized given the lack of clear criteria to guide its use.

Ongoing multicenter collaborative efforts such as the Transatlantic Australasian Retroperitoneal Sarcoma Working Group (TARPSWG) and CanSaRCC (Canadian Sarcoma Research and Clinical Collaboration) are helpful. Similar initiatives as well as the establishment of shared database using high-quality clinical data with connections through the Connective Tissue Oncology Society (CTOS) may facilitate the accumulation of knowledge and contribute to the generation of stronger evidence to direct management of mesenchymal tumors. Moreover, such efforts are paramount for ultra-rare tumors such as PEComa.

## Figures and Tables

**Table 1:** Summary of characteristics of PEComa cases treated with neoadjuvant RT.

Neoadjuvant Radiotherapy							
Author	Patient Information	Diagnosis	Location	Characteristics	Treatment	Follow-Up (Months)	Outcome
Weinreb et al. (2007) [20]	68 y/o male	Malignant PEComa	Thigh	7.8 cm; necrosis, high mitotic activity (42/10 HPFs), dedifferentiated patternless sarcoma areas	NR + resection	11	NED
Osei et al. (2007) [21]	49 y/o female	Malignant PEComa	Back/right shoulder	5.3 × 4.7 cm; undifferentiated and pleomorphic cells, necrosis	NC + NR + resection, 80% of tumor reduction after NC (doxorubicin and ifosfamide), size increase after NR	13	Lung metastasis
Yamashita et al. (2010) [22]	39 y/o female	Malignant PEComa	Right proximal tibia	6.5 cm; prominent hypercellularity with high mitotic activity	NR + resection	34	NED
Kirste et al. (2018) [23]	52 y/o female	Malignant PEComa	Liver	1280 cm^3^ (around 10 cm in diameter); high mitotic activity, nuclear pleomorphism, multinuclear giant cells and lack of necrosis	NR with 49% of tumor reduction + resection	21	NED
Saluja et al. (2018) Neoadjuvant [24]	28 y/o female	Malignant Pecoma	Oropharynx	7.2 × 5.1 × 2.9 cm; high mitotic activity, nuclear atypia, necrosis	NC − 1 mo (mTORi, everolimus − 1 mo, then Adriamycin + Ifosfamide − 1 mo) + NR (palliative dose) + resection	6	NED

**Table 2:** Summary of characteristics of PEComa cases treated with adjuvant RT.

Adjuvant Radiotherapy							
Author	Patient Information	Diagnosis	Location	Characteristics	Treatment	Follow-Up (Months)	Outcome
Folpe et al. (2000) [34]	21 y/o male	Malignant PEComa	Falciform ligament/ligamentum teres	20 cm; low mitotic activity, infiltrative growth, no necrosis, no vascular invasion	Resection + AR	3	Lung metastases
Folpe et al. (2005) [5]	71 y/o male	Malignant PEComa	Forearm	9 cm; high mitotic activity, high nuclear grade, mixed morphology with multinucleated giant cells (MNGCs)	Resection + AR	10	NED
Folpe et al. (2005) [5]	48 y/o female	PEComa with UMP	Cervix	2 cm; high nuclear grade, low mitotic activity, no necrosis, no vascular invasion, epithelioid and MNGC histology	Resection + AR	21	NED
Folpe et al. (2005) [5]	77 y/o female	PEComa with UMP	Neck	2.6 cm; low mitotic activity, mixed histology and MNGC, no necrosis, no vascular invasion	Resection + AR	6	NED
Folpe et al. (2005) [5]	56 y/o female	Malignant PEComa	Uterus	9 cm; high cellularity, high nuclear grade, high mitotic activity, atypia, no necrosis, no vascular invasion	Resection + AR + AC	11	Lung and bone metastases
Hornick et al. (2008) [36]	50 y/o female	Malignant PEComa	Retroperitoneum	13 cm; pleomorphic morphology, with marked nuclear atypia, high mitotic activity (22/10 HPF), necrosis	Resection + AR (for positive margins)	39	Lung, liver, abdominal wall metastases
Fukunaga et al. (2005) [35]	40 y/o female	Malignant PEComa	Uterus	30 × 27 × 18 cm and local metastases; high mitotic activity, vascular invasion, moderate atypia, necrosis	Resection + AC, AR	16	Died from multiple metastases
Silva et al. (2004) [33]	76 y/o female	Malignant PEComa	Uterus	Size was not reported; necrosis, atypia, high mitotic rate	Resection + AR	8	NED
Vang et al. (2002) [39]	75 y/o female	PEComa with UMP	Uterus	5 cm; absence of necrosis, mitoses, pleomorphism, vascular invasion	Resection + AR	30	NED
Jeon et al. (2005) [29]	9 y/o female	Malignant PEComa	Uterus	6.5 × 5 × 3.5 cm; no necrosis, mitoses but polymorphism and metastasis to one of the regional LN	NC (VID) + resection +AC (VID) + AR (45 Gy)	18	NED
Fink et al. (2004) [27]	51 y/o female	Malignant PEComa	Broad ligamentum	17 cm; pleomorphism, extensive necrosis and hemorrhages	Resection + AR (50.4 Gy).	15	NED
Lai et al. (2012) [37]	59-y/o male	Malignant PEComa	Mesenteric	9 × 11 cm; high-grade nuclear atypia, necrosis, vascular invasion, and high mitotic activity	Resection + adjuvant concurrent CRT (IA + 60 Gy)	6	Recurrence
Cole et al. (2021) [25]	42 y/o female	Malignant PEComa	Skin (cutaneous form)	3.5 cm; high mitotic activity, pleomorphism, nuclear atypia and hypercellularity	Resection + AR (60 Gy)	10	NED
Greveling et al. (2013) [28]	44 y/o male	Malignant PEComa	Cheek/cutaneous.	1 cm; high mitotic activity, significant nuclear polymorphism, no necrosis	Resection + AR	24	NED
Elousrouti et al. (2023) [26]	92 y/o female	Malignant PEComa	Skin/cutaneous form.	7 × 5.5 × 5 cm; nuclear polymorphism, atypia, high mitotic activity, no necrosis, no vascular invasion	Resection + AR + AC (mTORi)	6	NED
Akay et al. (2024) [30]	24 y/o female	Malignant PEComa	Adrenal gland	10 cm; necrosis, nuclear pleomorphism, high mitotic activity	Resection + AR (for positive margins) (46.8 Gy/26 fractions)	17	NED
Komune et al. (2020) [31]	24 y/o female	Malignant PEComa	Jugular foramen/neck	5 cm; infiltrative growth, vascular invasion, nuclear atypia, and high mitotic activity	Resection + AR	12	NED
Liu et al. (2019) [32]	80 y/o female	Malignant PEComa	Pelvis	10 cm; no microscopy reported	Resection + re-resection for recurrence in 1 month + AR + AC (mTORi)	50	NED
Liu et al. (2019) [32]	51 y/o female	Malignant PEComa	Pelvis	8.3 cm; high mitotic rate, necrosis	Resection + AR + AC (mTORi)	7	NED
Papoutsis et al. (2019) [38]	67 y/o female	PEcoma with UMP	Cervix	4.5 cm × 2.5 cm; modest nuclear polymorphism, low mitotic activity	Surgery (positive margins) + AR + AC	12	NED

**Table 3:** Summary of characteristics of PEComa cases treated with palliative RT.

Palliative Radiotherapy for Metastatic Disease or for PEComa Not Amenable to Definitive Resection							
Author	Patient Information	Diagnosis	Location	Characteristics	Treatment	Follow-Up (Months)	Outcome
Bonetti et al. (2001) [41]	19 y/o female	Malignant PEComa	Uterine	5.5 cm; necrosis, nuclear atypia, polymorphism and lymphovascular invasion, rare mitotic figures	Resection, recurrence in 1 month, another resection, inoperable residual disease, chemotherapy (IA) + consolidative RT to the pelvic mass for residual disease	18	Multiple metastases + local progression by 10th month
De León et al. (2010) [42]	76 y/o female	Malignant PEComa	Retroperitoneum + vaginal canal + metastases to sacrum	15 × 15 cm and 4 × 4 cm; necrosis, mild atypia, atypical mitoses	Primary resection, then RT (13 Gy) for sacrum metastasis	48	Multiple metastases to brain/spine
De León et al. (2010) [42]	38 y/o female	Malignant PEComa	Spine metastases, unknown primary site	Extensive necrosis, mild atypia, atypical mitoses	Palliative RT (13 Gy) for lumbar/sacral metastases	18	Multiple metastases to liver, lungs, spine by 11 months post-RT for spinal metastases
Lao W et al. (2015) [43]	47 y/o male	Malignant PEComa	Distal left femur + multiple lung metastases	5.2 × 3.2 × 2.6 cm; hypercellularity, nuclear atypia, atypical mitoses, necrosis	Palliative RT and systemic therapy; no details were reported. No RT site was reported (but likely distal femur given clinical description)	42	Alive at 42 months (no progression reported)
Bajaj, A et al. (2021) [40]	67 y/o male	Malignant PEComa	Lung (primary site) + metastases to ipsilateral lung, mediastinal, hilar nodes, ipsilateral malignant pleural effusion, chest wall, adrenal gland, etc.	5.8 × 5 cm (primary lung mass); high-grade, abundant necrosis	RT to the left lung mass (24 Gy + 12 Gy) + mTORi	4	Resolution of left lung mass, 2 new metastases in extrapulmonary sites
Wang et al. (2023) [44]	63 y/o female	Malignant PEComa	Primary uterine, recurrent pelvic mass + metastases to spine and lungs	10 × 10 cm (primary lesion), 8 × 4 cm (first recurrence); high grade, necrosis, infiltrative growth, nuclear atypia	Primary resection, resection of the recurred pelvic mass, second recurrence, SBRT 30 Gy/6 fractions delivered during cycles 1 and 4 of a 4-cycle (q3w) anti-PD-1 (Tislelizumab on Day8) + GM-CSF regimen (Day 1–7). GM-CSF concurrent; immunotherapy sequential Subsequent SBRT 24 Gy/6 fractions delivered to spinal metastasis within the same anti-PD-1 + GM-CSF regimen	4.3 (since progression in 2022—second recurrence)	Significant shrinkage of the recurred pelvic mass, stable spine metastasis post RT
Alnajar et al. (2018) [45]	44 y/o male	Malignant PEComa	Popliteal Fossa + metastases to spine	8.3 cm; nuclear atypia, necrosis, rare mitotic figures	Resection + systemic therapy (Pazopanib + Nivolumab) + palliative RT to spine metastases	12	Stable disease
Ross et al. (2011) [46]	46 y/o Female	Malignant Pecoma	Pelvis (recurrent, non-operable)	10 × 10 cm; pleomorphism, scattered mitoses, necrosis	Palliative RT + mTORi	37 (estimated from post-palliative RT based on provided timelines)	Stable disease with a complex clinical course; partial local response to palliative RT leading to resolution of right-sided hydronephrosis, subsequently, tumor size reduction with sirolimus (53 mm from 92 mm)

## Data Availability

No new data were created or analyzed in this study. Data sharing is not applicable to this article.

## Abbreviations

The following abbreviations are used in this manuscript:

AC	adjuvant chemotherapy
ACR	adjuvant chemoradiotherapy
AR	adjuvant radiotherapy
CRT	chemoradiotherapy
CSF	colony-stimulating factor
Gy	Gray
HPF	high-power field
IA	Ifosfamide, Adriamycin
LN	lymph node
mTORi	mammalian target of rapamycin inhibitor
NC	neoadjuvant chemotherapy
NED	no evidence of disease
NR	neoadjuvant radiotherapy
PEComa	perivascular epithelioid cell tumor
RT	radiotherapy
SBRT	stereotactic body radiotherapy
UMP	uncertain malignant potential
VID	vincristine–ifosfamide–doxorubicin regimen

## Appendix A

**Table A1:** Summary of RT treatments and clinical characteristics by treatment intent.

	Total Cases	Main Sites	Median Age	Females %	Males %	Mean Follow-Up (Months)	PEComa Cases with Positive Composite Descriptive Outcome (NED for Neoadjuvant/Adjuvant RT; for Palliative RT: Local Response and/or Local Stability and/or Systemic Disease Stability and/or Symptom Relief)
Neoadjuvant RT	5	Musculoskeletal 60%	49	80%	20%	17 (6–34)	4 (80%)
Adjuvant RT	20	Gynecological (including pelvis) 50%	51	80%	20%	16 (3–50)	15 (75%)
Palliative RT	8	Gynecological (including pelvis) 40%	46	62.5%	37.5%	23 (4–48)	4 (57%) *

* Lao et al. was excluded from outcome calculation because disease status was indeterminate (alive at 42 months without further clarification of response or progression).
